# Chronic suppurative otitis media due to *Streptomyces cacaoi*, the second case report in human infection

**DOI:** 10.1186/s12879-020-05222-0

**Published:** 2020-07-11

**Authors:** Lu Ai, Han Huang, Zhongwen Wu, Pingjuan Liu, Jianyu Huang, Yili Chen

**Affiliations:** Department of Laboratory Medicine, The First Affiliated Hospital of Sun Yat-sen University, Guangzhou, 510080 Guangdong China

**Keywords:** *Streptomyces cacaoi*, Chronic suppurative otitis media, Case report

## Abstract

**Background:**

*Streptomyces cacaoi*, Gram-positive, branched, filamentous bacillus forms without fragmentation, are saprophytic soil organisms rarely known to cause invasive infections other than mycetoma. Here we describe a case of chronic suppurative otitis media caused by *Streptomyces cacaoi* in a patient with hyperlipidemia in China.

**Case presentation:**

A 62-year-old female patient with hyperlipidemia suffered chronic suppurative otitis media caused by *Streptomyces cacaoi*. She had a favorable outcome with a 4-week course of ofloxacin ear drops.

**Conclusions:**

*Streptomyces cacaoi* is rarely reported to cause human infection. The introduction of molecular techniques improves the ability to identify rare species such as *Streptomyces* considerably. We report the case improve our ability to identify this pathogen and expand the range of known bacterial causes of human infection.

## Background

*Streptomyces cacaoi*, classified among the aerobic actinomycetes, presented Gram-positive, branched, filamentous bacillus forms without fragmentation with optimum growth at 35 °C [1]. *Streptomyces* are saprophytic soil organisms rarely known to cause invasive infections other than mycetoma. Ubiquitous nature and the low pathogenicity of *Streptomyces* organisms make most clinical isolates contaminants or colonizers [2, 3]. Until 2012 Gerald J. Pellegrini et al. first reported a scalp infection patient from Pondicherry, India and 16S rRNA sequence analysis provided the species identification [4]. Here we describe a case of chronic suppurative otitis media caused by *Streptomyces cacaoi* in a patient with hyperlipidemia in China, tending to improve our capacity to identify this isolate and widen the field of known bacterial causes of human infection.

## Case presentation

A 62-year-old woman presented to medical care (The First Affiliated Hospital of Sun Yat-sen University, Guangzhou, China) due to repeated purulence and decreased hearing of right ear for 30 years, and aggravated for 1 year. The accidentally insect flew into the right ear, and then purulence and decreased hearing developed and sustained for 30 years. The patient had not been treated with antibiotics for ear infection before. Besides, she had a history of hyperlipidemia and treated with traditional Chinese Medicine.

**Fig. 1:**
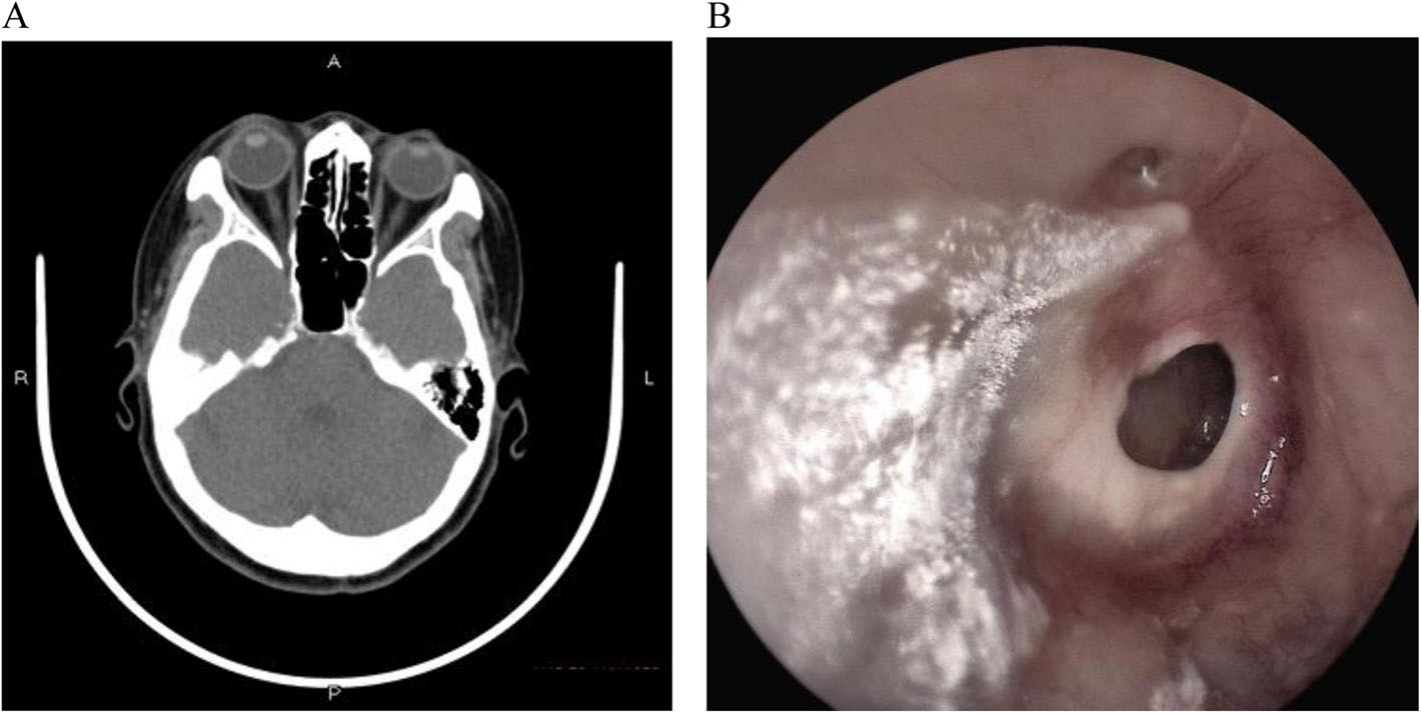
Computed tomography of mastoid process revealed chronic mastoiditis and tympanic membrane perforation of right side (**a**), and white aerial hyphae can be seen in the right external auditory canal, with large perforation in pars tensa of tympanic membrane in aural endoscopy (**b**)

Upon medical checkup, she had a tympanic temperature of 36.5 °C, blood pressure of 129/87 mmHg, pulse rate of 72/min and respiratory rate of 19/min. Computed tomography of mastoid process revealed chronic mastoiditis and tympanic membrane perforation of right side. Aural endoscopy showed that the left external auditory canal unobstructed and the tympanic membrane intact with local calcified plaques; white aerial hyphae can be seen in the right external auditory canal, with large perforation in pars tensa of tympanic membrane, and drum room clean (Fig. 1). Laboratory evaluation revealed leukocyte count of 6810/mm^3^ with 68.9% neutrophils and 23.7% band forms, total cholesterol 7.2 mmol/L (normal range, 3.1–5.7 mmol/L), high-density lipoprotein 2.02 mmol/L (normal range, 1.09–1.63 mmol/L), lipoprotein(a) 860 mg/L (normal range, 60-300 mg/L).

**Fig. 2:**
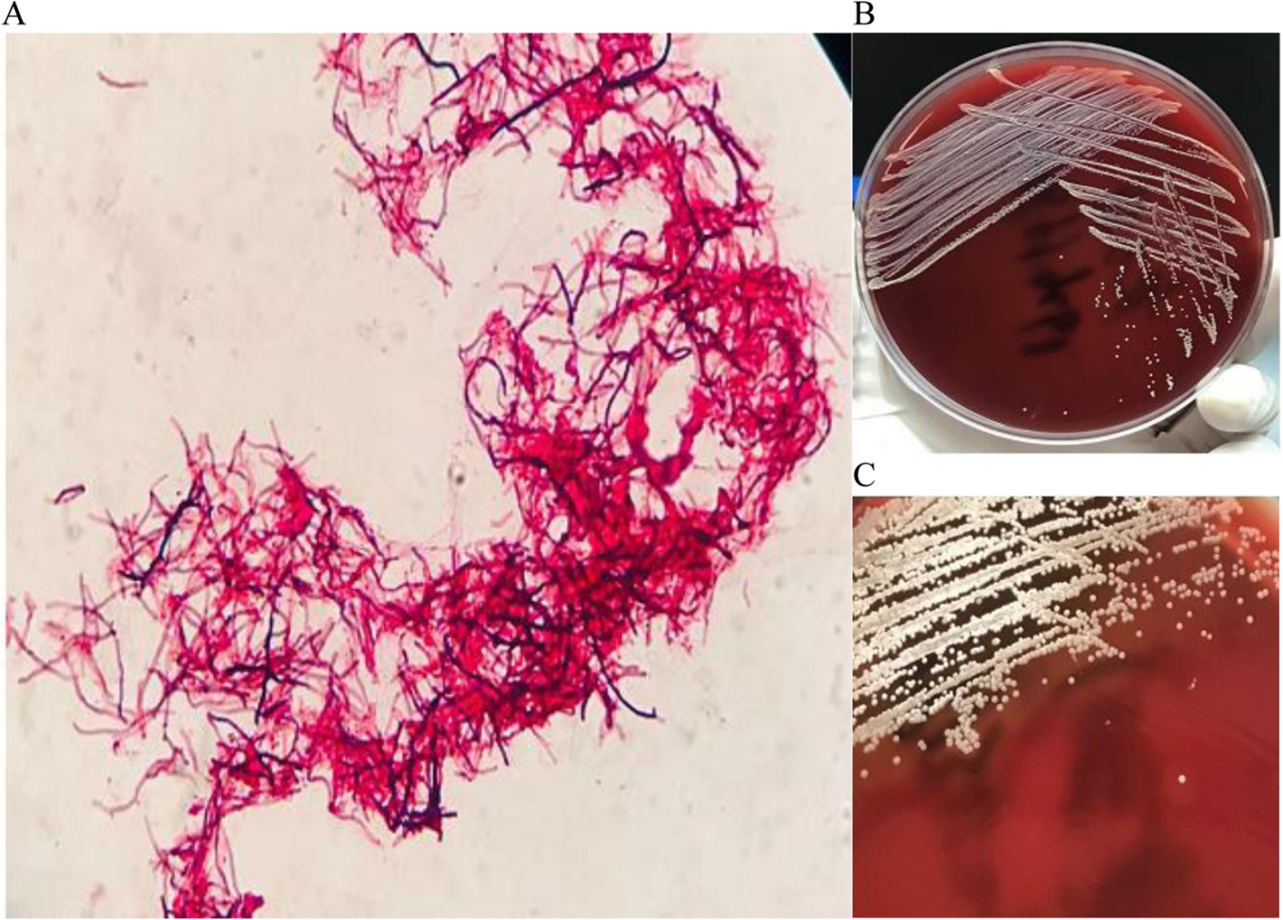
Gram stain of ear exudate, demonstrating Gram-positive, filamentous, non-spore-forming bacilli (**a**), and The organism appeared grayish white, dry, wrinkled small colonies biting agar after 24 h incubation on blood agar (**b**), beta hemolysis obvious after 48 h incubation (**c**)

On day 4, the patient underwent microscope supporting right ear tympanoplasty, ossicular chain release and aticoantrotomy under general anesthesia. Empiric antibiotic treatment with Cefuroxime sodium (1.5 g every day) were started and lasted 1 day for prevention of postoperative infection. Ear exudate intraoperative was collected and the Gram stain of the colonies demonstrated Gram-positive branched filamentous bacilli, with weak acid fast staining negative (Fig. 2a). The organism appeared grayish white, dry, wrinkled small colonies biting agar after 24 h incubation on blood agar, beta hemolysis obvious after 48 h incubation. The isolate exhibited distinctive powder or velvet colonies that developed characteristic white aerial hyphae after 72 h of aerobic growth (Fig. 2b, c). To confirm the identity of the isolate, we performed molecular identification using primer sets 16S-forward (5′AGAGTTTGATCCTGGCTCAG3′) and 16S-reverse (5′GGTTACCTTGTTACGACTT3′) by polymerase chain reaction amplification of the 16S rRNA gene. The sequence analysis of 1,450 bp nucleotide were queried against the GenBank 16S rRNA gene database and the best match returned was the *Streptomyces cacaoi* (GenBank accession number AB184115), with 99.2% similarity.

Due to the limitations of experimental conditions, the antibiotic susceptibility of the *Streptomyces cacaoi* strain was mostly determined by the Kirby-Bauer disk diffusion method on Mueller-Hinton agar plates. Although there are no categorical interpretative criteria for antimicrobial susceptibility testing data for *Streptomyces cacaoi*, the isolate inhibition zone (millimeter) for antimicrobials demonstrated: minocycline 20 mm, gentamicin 37 mm, clindamycin 6 mm, erythromycin 6 mm, sulphamethoxazole/trimethoprim 6 mm, amikacin 32 mm, levofloxacin 6 mm, moxifloxacin 20 mm, ciprofloxacin 12 mm, linezolid 50 mm, cefotaxime 6 mm, ceftriaxone 6 mm, cefepime 6 mm. Moreover MIC testing indicated resistance to amoxicillin-clavulanic acid (> 256 μg/ml), meropenem(> 32 μg/ml), and intermediate resistance observed to imipenem (6 μg/ml) with the employ of Etest (Biomerieux SA, Marcy, France).

**Fig. 3:**
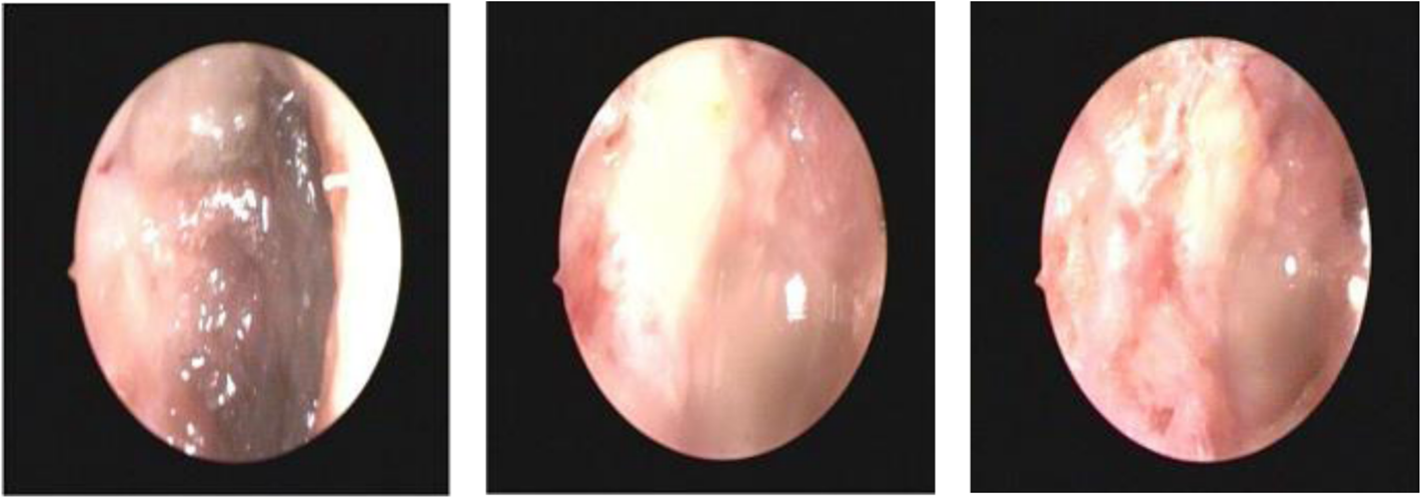
The aural endoscope indicated intact tympanic membrane after operation

With external application of ofloxacin ear drops sustained for 2 weeks after surgery, the patient returned for pure tone audiometry and ear endoscopy and recovered in good condition. One month later she accepted cleaning of the right external auditory canal which was packed with ear dressing under the aural endoscope and was delighted to see the intact tympanic membrane after operation (Fig. 3).

## Discussion and conclusions

The *Streptomyces cacaoi*, which was discovered and isolated from cacao beans, was primitively described by Waksman in 1932, corrected by Waksman and Henrici in 1948 [5], and at last revised by Lanoot et al. as *S. Cacaoi* in 2002 [6]. The organism, classified among the aerobic actinomycetes, presented Gram-positive, branched, filamentous bacillus forms without fragmentation with optimum growth at 35 °C. The organism stained acid fast negative by the modified Kinyoun method, which is different from other members of the group, such as *Nocardia spp*. And *Rhodococcus spp.*.

The *Streptomycetes* are classified as a separate genus within the aerobic actinomycetes, most well known for the approximately 600 different species [3]. Ubiquitous nature and the low pathogenicity of *Streptomyces* organisms make most clinical isolates pollutants or colonizers [7, 8]. For it is difficult to determine the pathogenicity of the omnipresent *Streptomyces* definitively, several guiding principles have been given to assist the diagnosis of actual infection: isolate from sterile site, morphological identification under direct microscope, and eliminate other pathogeny by Kapadia et al. [9]. A stringent clinical and microbiologic correlation is needed in this regard, such as clinical manifestations, isolation of the organism from sterile sources (ideally in large quantities), direct microscopic identification in infected tissue [10]. Direct specimen microscopy is extremely important, and many times extended incubation time can prove effective [6]. In this case, white aerial hyphae observed by aural endoscopy initially made us confused whether it was fungus otitis media or suppurative otitis media, but the morphology observed by the microscope gave some effective clues. Though the morphology changed with the strains and their growth stages in infected tissues, bacterial culture remains the gold standard for final etiological diagnosis to date. On culturing, *Streptomyces cacaoi* produces dry, chalky, gray-white colonies that emit pungent, musty odor or earthy scent [11].

In the last decade, owing to the extensive use of PCR and DNA sequencing identification to the species level has evolved to more polyphasic approach, in the meantime 16S rDNA sequencing has played a critical role in more accurate identification of bacterial isolates and the discovery of novel bacteria in clinical microbiology laboratories [12]. For the identification of bacteria with unusual phenotypic profiles, emergent bacteria, slow growth bacteria, uncultivable bacteria and culture-negative infections, 16S rDNA sequencing make a big difference [11]. We can reasonably speculate that previously similar *Streptomyces* infections were under diagnosed on account of technical and methodological limitations, the lack of molecular techniques included.

The susceptibility of the *Streptomyces cacaoi* to different antimicrobials was performed according to Clinical and Laboratory Standards Institute guidelines M24-A2 using broth micro dilution method [13]. The currently recommended experience therapy for *Streptomyces* actinomycetoma infection is trimethoprim-sulfamethoxazole which is used worldwide. But in an extensive investigate of 92 isolates by Rose et al., the susceptibility testing results stated a critical degree of resistance to many antibiotics: 79% of strains were resistant to ceftriaxone, 75% to sulfamethoxazole and to erythromycin, 65% to trimethoprim-sulfamethoxazole, 54% to ciprofloxacin, and all were sensitive to amikacin and linezolid [14]. It suggested that the fusidic acid, amikacin, novobiocin, doxycycline, gentamicin, and linezolid combination with trimethoprim-sulfamethoxazole should be reconsidered. Meanwhile the fact that Streptomyces spp. are slow-growing bacteria renders prolongation of antibacterial treatment necessary [15].

To our knowledge, this is the second report of *Streptomyces cacaoi* human infection. As the experience limited, we need further research to better understand the isolates of the genus *Streptomyces*: the predisposing factors for infection, and the process, treatment, and evolution of these infections. On the other hand, the introduction of molecular techniques applied to a bacterial isolate has brought about a vast improvement in the section of identify rare species, especially in a taxon such as *Streptomyces*, which consists of approximately 600 different species.

## Data Availability

Data and materials of this report are publicly available from the corresponding author on reasonable request.

## Abbreviations

*S. Cacaoi*: *Streptomyces Cacaoi*
PCR: Polymerase Chain Reaction
DNA: Deoxyribonucleic Acid
rDNA: ribosome Deoxyribonucleic Acid
